# Emergency Department Encounters Preceding Hip Fracture in Older Adults: A Window for Future Risk Identification

**DOI:** 10.1016/j.acepjo.2026.100498

**Published:** 2026-09-03

**Authors:** Ghada Elhadari, Hani Mowafi

**Affiliations:** Department of Emergency Medicine, Yale University, New Haven, Connecticut, USA

## Abstract

**Objectives:**

To describe the frequency of prior emergency department (ED) encounters before hip-fracture encounters among older adults in the United States (US).

**Methods:**

This was a retrospective descriptive study of US older adults aged 65 to 110 years (January 1, 2016 to December 31, 2025) using Epic Cosmos. We identified hip-fracture encounters and evaluated the proportion with an ED encounter in the preceding 12 months, overall and stratified by demographic characteristics.

**Results:**

Among 1,873,342 hip fracture-encounters representing 1,355,249 distinct patients, 37.2% had ≥ 1 ED encounter in the prior 12 months. This proportion increased with age: (≥65 to <75, 34.7%; ≥75 to <85, 36.8%; ≥85, 39.4%). Proportions slightly varied by sex (women 36.7% vs men 38.5%, and other/unknown 21.0%), by ethnicity (Hispanic/Latino 35.5%, non-Hispanic/Latino 38.1%, nonclassified 31.7%), and by region (37.5% South, 32.6% West, 40.1% Midwest, 36.4% Northeast, and 36.6% in regions not otherwise classified).

**Conclusion:**

ED encounters frequently preceded older adults’ hip fractures. These findings may help inform future studies examining opportunities for earlier identification of vulnerable older adults.

**The Bottom Line**

Can emergency department (ED) encounters help identify opportunities to prevent hip fractures in older adults? This study examined how often hip-fracture encounters among older adults were preceded by an ED encounter in the prior year. Using a national database, we identified over 1.8 million hip-fracture encounters among adults aged ≥65 years between 2016 and 2025. Overall, 37.2% of hip-fracture encounters were preceded by at least one ED encounter within the prior year. These findings suggest that ED encounters commonly occur before hip fracture and may represent opportunities to identify older adults at increased fracture risk.

## 1 Introduction

### 1.1 Background

Analysis of the Global Burden of Disease data in 2019 demonstrated the continuing significance of the global burden of hip fractures, with an increase in incidence among patients aged ≥55 years during the past 3 decades.^1^ Data from the 2019 Healthcare Cost and Utilization Project and National Vital Statistics System reported 318,797 emergency department (ED) visits, 290,130 hospitalizations, and 7731 deaths related to hip fractures among older adults.^2^

Across 11 high-income countries, the utilization of health care services for an older person with frailty for 1 year before and after a hip fracture was substantially variable. When combined, France and the United States had the highest number of days in hospitals and rehabilitation facilities over the year. The United States was the most expensive country due to high prices and above-average utilization of postacute rehabilitation care.^3^

Effective hip fracture prevention is multifactorial, encompassing osteoporosis screening, pharmacological therapy, medication reconciliation, nutritional support, exercise-based fall prevention, environmental modification, and mechanical hip protection. The strongest evidence supports structured, coordinated care models—such as Fracture Liaison Services and orthogeriatric programs—over isolated, single-domain interventions. However, these programs are predominantly embedded in elective outpatient or postfracture inpatient pathways, leaving patients who present acutely to the ED largely outside the reach of systematic preventive care at a moment when intervention may still alter their trajectory.^4^, ^5^, ^6^, ^7^, ^8^, ^9^, ^10^, ^11^, ^12^, ^13^, ^14^, ^15^, ^16^, ^17^, ^18^, ^19^, ^20^, ^21^, ^22^, ^23^

### 1.2 Importance

Identification of individuals at high risk of hip fracture enables simple preventive measures to be taken, potentially reducing the likelihood of fracture occurring, which can significantly improve the quality of life and reduce health care costs.

Although the ED is traditionally focused on acute stabilization rather than preventive care, it has increasingly been recognized as a site for public health screening and risk stratification,^24^, ^25^, ^26^ particularly among older adults, who may have limited engagement with primary care.^27^

Prior single-center work has demonstrated that a substantial proportion of patients with hip fractures have a documented acute care encounter in the year preceding their fracture, representing a potential missed opportunity for preventive intervention; 45.5% of patients with hip fracture had a preceding encounter, yet only 8% of those presenting for a fall received falls counseling.^28^ However, this finding has not been examined at scale across a large patient population.

Epic Cosmos is a data set created in collaboration with a community of health systems using Epic and includes >300 million patients.^29^

### 1.3 Goal of the Investigation

This study aimed to examine the proportion of older adults with hip fracture who had at least one ED encounter within the preceding 12 months using the Epic Cosmos national database and SlicerDicer analytic tool. We additionally examined this proportion across demographic characteristics including age, sex, ethnicity, insurance status, and geographic region.

## 2 Methods

We conducted a retrospective, descriptive study of older adults aged ≥ 65 and ≤ 110 years receiving care in the United States from January 1, 2016 to December 31, 2025, using Epic Cosmos via the SlicerDicer analytic tool.

Epic Cosmos is a deidentified electronic health record data set created in collaboration with a community of health systems using Epic. The data set contains longitudinal records and integrates both inpatient and outpatient charts into a single, comprehensive record—including as patients move between health systems. At the time of access, Cosmos included data from > 300 million patients, > 20 billion encounters, > 2000 hospitals, and > 47,000 clinics.^29^ Because the number of contributing sites within Epic Cosmos changes over time, we assessed the stability of the hip fracture-encounter denominator by calculating hip-fracture encounters as a percentage of total encounters among adults aged ≥65 in the United States, by calendar year.

SlicerDicer is an analytic interface within Epic that allows users to perform aggregated queries and descriptive analyses of the Cosmos data set. Given platform constraints, analyses were designed to identify clinically meaningful patterns using aggregated encounter-level data.

### 2.1 Study Cohort

We queried Cosmos to identify adults aged ≥ 65 and ≤ 110 years with any encounter type associated with a hip fracture diagnosis. We defined hip fracture using International Classification of Diseases, 10th Revision codes S72.0–S72.2, consistent with prior administrative data definitions and validation studies.^30^, ^31^, ^32^ To reduce inclusion of repeat or follow-up fracture-related encounters, we excluded encounters preceded by another encounter of any type with a hip fracture diagnosis within the prior 12 months. We then used the SlicerDicer Measure function to calculate the proportion of qualifying hip-fracture encounters that were preceded by at least 1 separate ED encounter, for any diagnosis, within the prior 12 months, using the platform’s temporal "preceded by" relationship between encounters.

The primary outcome was the proportion of qualifying hip-fracture encounters that were preceded by at least 1 ED encounter within the preceding 12 months. We conducted secondary descriptive analyses to examine this proportion across demographic subgroups, including age group, sex, race/ethnicity, insurance status, geographic region, and calendar year.

### 2.2 Statistical Analysis

Analyses were descriptive and reported as counts and percentages. Proportions were calculated using the total number of hip-fracture encounters overall, or within each subgroup for stratified analyses, as the denominator. No inferential statistical testing or multivariable modeling was performed, consistent with the exploratory and proof-of-concept nature of the study.

## 3 Results

During the 10-year study period, 1,873,342 hip-fracture encounters among adults aged ≥65 and ≤110 years were identified, representing 1,355,249 distinct patients. Among these encounters, 697,756 (37.2%) were preceded by at least 1 ED encounter in the prior 12 months. This proportion increased over the study period, from 32.1% in 2016 to 41.2% in 2025 (Supplementary Table 1). A similar increase was observed in the comparison population of older adults without a hip-fracture diagnosis, from 24.8% to 36.4% for the same period (Supplementary Table 2). The proportion of encounters with a hip-fracture diagnosis from all older adults’ encounters (Supplementary Table 3) remained stable at 0.03% to 0.04% throughout 2016–2025. For comparison, 32.7% of older-adult encounters, excluding those with a hip fracture diagnosis, during the same period, were preceded by an ED encounter within 12 months, versus 37.0% in the hip-fracture cohort.

The proportion with a prior ED encounters increased with age: 34.7% for ages ≥65 to <75, 36.8% for ages ≥75 to <85, and 39.4% for ages ≥85 years. Prior ED encounters were observed in 36.7% of women and 38.5% of men, and 21.0% among those with other/unknown sex. By ethnicity, 35.5% of Hispanic or Latino patients, 38.1% of non-Hispanic or Latino patients, and 31.7% of those with ethnicity not otherwise classified had a prior ED encounter.

By US region, prior ED encounters occurred in 37.5% of hip-fracture encounters in the South, 32.6% in the West, 40.1% in the Midwest, 36.4% in the Northeast, and 36.6% among those with region not otherwise classified. Encounters could include ≥1 option of race and insurance categories; therefore, they are not summarized as mutually exclusive totals. (Table).

**Table:** Hip-fracture encounters preceded by an ED encounter within the prior 12 months.

Group	Hip fracture N	Prior ED encounter n (%)^b^
Overall	1,873,342	697,756 (37.2)
Age Group		
≥ 65 and < 75 y	478,345	161,055 (34.7)
≥ 75 and < 85 y	693,599	255,125 (36.8)
85 y or more	701,398	276,576 (39.4)
Sex		
Woman	1,292,083	474,250 (36.7)
Man	581,112	223,474 (38.5)
Other/unknown	138	29 (21.0)
Race^a^		
American Indian or Alaska Native	12,118	4,576 (37.8)
Asian	35,586	10,732 (30.1)
Black or African American	91,775	42,076 (45.9)
Native Hawaiian or other Pacific Islander	4000	1515 (37.9)
White	1,548,438	584,041 (37.7)
Other	324,889	112,381 (34.6)
Ethnicity		
Hispanic or Latino	60,325	21,404 (35.5)
Not Hispanic or Latino	1,597,972	608,289 (38.1)
None of the above	215,045	68,063 (31.7)
US Region		
South	715,759	268,278 (37.5)
West	270,588	88,323 (32.6)
Midwest	487,553	195,703 (40.1)
Northeast	379,045	137,989 (36.4)
None of the above	20,397	7463 (36.6)
Insurance^a^		
Medicaid	204,112	92,105 (45.1)
Medicare	1,172,067	444,481 (37.9)
Self-pay	3199	472 (14.8)
Other	1,505,204	556,737 (37.0)

a Encounters could include more than one option.

b Percentages represent the proportion of hip-fracture encounters within each subgroup that had at least one ED encounter in the preceding 12 months.

## 4 Limitations

The study has several limitations related to the use of an aggregated EHR-based tool for analysis. These limitations were considered in the interpretation of findings.

• This was descriptive and retrospective, and we did not perform adjustment for confounding; observed differences across subgroups may reflect underlying case-mix rather than independent associations.
• Epic Cosmos is composed of participating organizations contributing longitudinal clinical data. The network continues to expand over time, and contributing organizations may vary across study years, which should be considered when interpreting temporal trends. However, the proportion of hip-fracture encounters relative to total encounter volume remained stable (0.03% to 0.04%; Supplementary Table 3). This does not eliminate the potential influence of changes in the composition of the Epic Cosmos network over time.
• Cosmos Slicer Dicer provides aggregated encounter-level outputs and does not provide patient-level data for analysis; encounter-level analyses may not fully represent unique patients, and more granular longitudinal analyses, including characterization of preceding ED encounters (eg, common diagnoses or chief complaints), were not possible. Temporal sequencing is based on encounter-level data and may not fully distinguish incident hip fractures from follow-up or repeat fracture-related encounters. To mitigate this and reduce inclusion of repeat fracture-related episodes, we applied available sequence-based exclusions for prior hip fracture-coded encounters within the preceding 12 months.
• Our outcomes reflect hip fracture diagnoses captured within participating health systems; fractures treated elsewhere would not be observed.

## 5 Discussion

In this study, 37.2% of hip-fracture encounters among older adults were preceded by at least 1 ED encounter in the preceding year. These findings suggest that ED encounters commonly occur before hip fracture among older adults. Although this relationship should not be interpreted as causal, the frequency with which ED encounters preceded hip fracture suggests that EDs may warrant further investigation as potential settings for future risk stratification and preventive interventions.

Both the hip-fracture cohort and the comparison population showed a similar rising trend in the proportion preceded by an ED encounter, suggesting that the increase was not unique to the hip fracture cohort and may reflect broader temporal changes in health care utilization.

The proportion of hip-fracture encounters preceded by an ED encounter (37.2%) was modestly higher than the comparable proportion among older adults with encounters unrelated to hip fracture (32.7%). This difference may reflect underlying frailty or comorbidity common to both ED utilization and fracture risk, a genuine, missed opportunity for preventive intervention, or some combination of both; a rigorously matched comparison cohort using patient-level data would help distinguish between these explanations in future work.

Hip fractures in older adults are strongly associated with mortality and long-term morbidity, with loss of independence, functional decline, and high health system costs. Large observational studies and reviews have consistently shown increased short- and long-term mortality after hip fracture as well as residual disability and persistent decrease in mobility and ability to perform daily living activities among survivors.^33^^,^^34^ These findings underscore the importance of prevention efforts for hip fractures, given the substantial clinical and public health implications.^1^

The observation that many hip-fracture encounters among older adults had preceding ED encounters suggests that ED utilization before hip fracture may reflect broader vulnerability rather than isolated fracture-specific risk factors. This concept aligns with geriatric syndromes as shared-risk, multifactorial conditions in which multiple interacting factors contribute to adverse outcomes. From this perspective, preceding ED encounters may reflect underlying health status, health care utilization patterns, or other shared factors associated with both ED use and hip fracture and may represent markers of underlying vulnerability that warrant further investigation rather than simply isolated health care interactions.

There are effective interventions that already exist for preventing falls and fractures, making these results clinically relevant and highlighting the importance of identifying opportunities for early prevention. Medication risk reduction, strength and balance training, improving visual acuity, and addressing home hazards reduce fall rates and fall-related injuries. Fracture risk can be reduced by osteoporosis evaluation and treatment; bisphosphonates and other antiresorption/anabolic therapies, shared decision-making for pharmacologic interventions and nonpharmacologic strategies, and postfracture “fracture liaison services”.^17^^,^^23^^,^^35^, ^36^, ^37^ There is a substantial implementation gap, as osteoporosis frequently goes underdiagnosed and undertreated, even after sentinel frailty events.

ED involvement would not necessarily mean that the comprehensive intervention would happen during the acute encounter itself; identification of at-risk patients and facilitating referral or communication with primary care physicians, geriatrics services, physical therapy, or other outpatient resources may represent more feasible approaches, that deserve further investigation.^38^^,^^39^

From a health systems perspective, many older adults with medically complex conditions seek care in the ED, including those with limited access to longitudinal primary care. The ED may serve as a potential efficient point for identifying high-risk patients, who would otherwise not receive timely fall-risk assessment or osteoporosis evaluation.^40^, ^41^, ^42^ However, not all older adults presenting to the ED are at equal risk of subsequent hip fracture, and broad implementation of prevention interventions across all ED patients would likely be impractical. Future work should therefore focus on developing and validating ED-feasible risk stratification approaches that can identify patients most likely to benefit from targeted prevention efforts. Furthermore, the ED setting is constrained with high flow, competing priorities, and limited follow-up. Feasible strategies need to be brief and automated, such as EHR-driven triggers and prompts for high-risk profiles; automated referral to geriatrics/primary care/physical therapy; standardized discharge instructions; medication risk alerts; or linkage to community fall prevention programs.

Prior literature demonstrates that old age and falls are risk factors for hip fracture, and population-level hip fracture epidemiology is well described.^1^ However, the frequency with which ED encounters precede hip fractures has been less commonly quantified at scale. In a single-center study, Pierrie et al found that nearly half of patients with hip fracture had an ED visit or hospital admission in the preceding year, but few patients presenting after a fall received falls counseling, highlighting that acute care encounters may represent underused opportunities for fracture prevention.^43^ Our study extends the literature by describing the frequency of prior ED use among older adults with hip fracture, using a large national database. This encounter-based assessment may be useful for operational planning, can help estimate the volume of patients who may be relevant when designing future prevention strategies, and help inform future studies examining whether EDs can contribute to risk stratification, prediction, and preventive strategies for this population.

## 6 Implications and Future Directions

Future studies should evaluate whether ED encounters can be leveraged for risk stratification and whether ED-appropriate prediction models can identify older adults at increased risk for subsequent hip fracture. Additional work should develop and validate ED-feasible hip fracture risk stratification and near-term prediction models to support near-term intervention. Given the burden of hip fractures and the frequency of ED contact among older adults, interventions that effectively leverage ED encounters could represent a scalable approach to reducing preventable fractures.

## Author Contributions

GE contributed to conceptualization, data curation, formal analysis, visualization, and writing of the original draft. HM contributed to conceptualization, methodology, supervision, validation, and critical review, and editing of the manuscript.

## Funding and Support

By *JACEP Open* policy, all authors are required to disclose any and all commercial, financial, and other relationships in any way related to the subject of this article as per ICMJE conflict of interest guidelines (see www.icmje.org). The authors have stated that no such relationships exist.

## Conflict of Interest

All authors have affirmed they have no conflicts of interest to declare.
