# Supplementary material for: Emergency Department Encounters Preceding Hip Fracture in Older Adults: A Window for Future Risk Identification
Source: J Am Coll Emerg Physicians Open. 2026 Sep 3;7(5):100498. doi: 10.1016/j.acepjo.2026.100498 (PMC13571583; doi:10.1016/j.acepjo.2026.100498)
Supplement: Supplementary file 1 [file mmc1.docx]

**Supplementary Table 1.** Proportion of hip fracture encounters preceded by an emergency encounter within the prior 12 months, among adults aged ≥65 in the United States, by calendar year, 2016–2025.

| **Year** | **Hip Fracture encounter** | **Hip fracture encounter preceded by ED encounter** |
| --- | --- | --- |
| 2016 | 92,519 | 29,707 (32.1%) |
| 2017 | 117,029 | 38,614 (33.0%) |
| 2018 | 139,722 | 48,809 (34.9%) |
| 2019 | 156,734 | 56,757 (36.2%) |
| 2020 | 160,164 | 57,821 (36.1%) |
| 2021 | 187,947 | 64,314 (34.2%) |
| 2022 | 216,764 | 79,245 (36.6%) |
| 2023 | 239,526 | 93,384 (39.0%) |
| 2024 | 266,905 | 107,102 (40.1%) |
| 2025 | 296,032 | 122,007 (41.1%) |

Hip-fracture encounters exclude those preceded by another hip-fracture-coded encounter for the same patient within the prior 12 months, consistent with the primary cohort definition (Supplementary Table 1). 'Preceded by ED' indicates an ED encounter for the same patient in the 12 months prior to the hip-fracture encounter.

**Supplementary Table 2.** Proportion of encounters without a hip-fracture diagnosis preceded by an ED encounter within the prior 12 months, among adults aged ≥65 in the United States, by calendar year, 2016–2025.

| **Year** | **Comparison cohort (encounters other than hip fracture encounters N** | **Comparison cohort encounters preceded by ED encounter n (%)** |
| --- | --- | --- |
| 2016 | 250,271,801 | 62,052,690 (24.8%) |
| 2017 | 312,375,076 | 84,030,385 (26.9%) |
| 2018 | 374,358,774 | 112,470,714 (30.0%) |
| 2019 | 439,513,568 | 139,321,150 (31.7%) |
| 2020 | 511,982,335 | 159,581,026 (31.2%) |
| 2021 | 634,258,057 | 190,324,670 (30.0%) |
| 2022 | 704,492,985 | 229,099,855 (32.5%) |
| 2023 | 790,158,614 | 272,319,130 (34.5%) |
| 2024 | 871,496,570 | 311,705,952 (35.8%) |
| 2025 | 996,042,876 | 362,796,631 (36.4%) |

The comparison cohort includes encounters among adults aged ≥65 years in the United States with no hip-fracture diagnosis code. The comparison cohort and the qualifying hip-fracture cohort shown in Supplementary Tables 1 and 3 were defined using different inclusion criteria and are not complementary categories; therefore, their encounter counts should not be summed to derive the total cohort size. Specifically, the comparison cohort excludes all hip-fracture-coded encounters, whereas Supplementary Table 2 includes only hip-fracture-coded encounters with no preceding hip-fracture-coded encounter within the prior 12 months.

**Supplementary Table 3.** Hip fracture encounters as a proportion of total encounters among adults aged ≥65 in the United States, by calendar year, 2016–2025.

| **Year** | **N. of older adults encounters** | **Hip fracture encounters n(%)** |
| --- | --- | --- |
| 2016 | 250,685,342 | 92,519 (0.04%) |
| 2017 | 312,865,302 | 117,029 (0.04%) |
| 2018 | 374,951,698 | 139,722 (0.04%) |
| 2019 | 440,217,934 | 156,734 (0.04%) |
| 2020 | 512,696,732 | 160,164 (0.03%) |
| 2021 | 635,093,062 | 187,947 (0.03%) |
| 2022 | 705,447,260 | 216,764 (0.03%) |
| 2023 | 791,257,930 | 239,526 (0.03%) |
| 2024 | 872,723,485 | 266,905 (0.03%) |
| 2025 | 997,538,418 | 296,032 (0.03%) |

Total encounters and hip-fracture encounters are restricted to adults aged ≥65 in the United States. Hip-fracture encounters exclude those preceded by another hip-fracture-coded encounter for the same patient within the prior 12 months.

Annual encounter counts should be interpreted in the context of the expanding Epic Cosmos network over the study period.
